# Cloning of the Quail *PIWI* Gene and Characterization of PIWI Binding to Small RNAs

**DOI:** 10.1371/journal.pone.0051724

**Published:** 2012-12-19

**Authors:** Rong Chen, Guobin Chang, Ying Zhang, Aiqin Dai, Teng Ma, Jianchao Li, Fei Zhai, Dengke Hua, Mingxiu Xia, Guohong Chen

**Affiliations:** College of Animal Science and Technology, Yangzhou University, Yangzhou, China; Medical College of Wisconsin, United States of America

## Abstract

The PIWI protein regulates gene expression at the epigenetic and post-transcriptional level with a variety of endogenous small non-coding RNAs. In poultry, the biological function of the PIWI protein and PIWI binding to small RNAs had not been determined. The present study cloned and analyzed the sequences of the PIWIL1 protein. We also characterized PIWIL1 binding to small RNAs from adult quail testis, where the PIWIL1 protein is specifically expressed. Small RNAs showed a strong peak at 24–27 nt in the testicular RNA library, mapped primarily to repeat sequences and were similar to rasiRNAs. MicroRNAs (miRNAs) were abundant in the ovarian RNA library at a peak of 22 nt.

## Introduction

The *PIWI* gene was first detected in Drosophila germline stem cells (GSCs) [1] and encodes a nucleoplasmic protein whose activity modulates the number and division rate of GSCs [2]. The AGO protein family contains the core components of the RNA-induced silencing complex (RISC) and participates in the RNA-guided gene silencing process [3]. In Drosophila, the PIWI subfamily contains three members: PIWI, AUB and AGO3. Female *aub* mutants give rise to abnormal eggs, and male mutants are sterile. Additionally, both male and female *piwi* mutants are sterile. In the germline, the three PIWI proteins combine with a class of repeat-associated small interfering RNAs (rasiRNAs) to exert splicing activity similar to RNase H. Thus, rasiRNAs are distinct from other RNAi pathways, such as miRNAs and siRNAs. In animals, miRNAs require Dicer-1 for their production and siRNAs require Dicer-2. However, rasiRNAs require neither Dicer-1 nor Dicer-2 [4]. Compared to miRNAs and siRNAs, rasiRNAs are longer (24–29 nt) [5]–[6]. Unlike miRNAs and siRNAs, which function through the AGO protein subfamily, rasiRNAs act through the PIWI protein subfamily [7]. In germline cells, rasiRNAs are involved in establishing and maintaining heterochromatin structure, transposons silencing and DNA damage suppression [8].

MIWI, MILI and MIWI2 are three murine PIWI proteins. Knockout of each protein led to male sterility through the suspension of spermatogenesis and apoptosis of germ cells [9]–[11]. However, female mutants were fertile and produced normal eggs. In mice, the PIWI/piRNA pathway is required for reproduction-related processes such as the asymmetric division and differentiation of germ stem cells, meiosis and spermatogenesis [12]–[15]. Given the nucleotide length, testicular specificity, PIWI protein interaction, genomic origin and 5′ end bias for uracil, these characteristics indicate that piRNAs are similar to mammalian rasiRNAs [16]. However, piRNAs are primarily derived from intergenic sequences and a single strand, while rasiRNAs are produced from bidirectional transcripts of repeat sequences [6]. In humans, the PIWI protein is involved in the formation and development of tumors in addition to spermatogenesis [17]–[18]. Thus, the similarities and differences between rasiRNAs and piRNAs require further study.

At present, the study of small RNAs in poultry is focused on miRNAs, including the construction of miRNA expression profiles for various tissues, the screening of tumor markers and the identification of reproduction-related miRNAs [19]–[22]. We previously reported our findings regarding the PIWI protein and PIWI binding to small RNAs [23]–[24]. Yang et al. also cloned 156 piRNA-like RNAs from adult chicken testis [25]. Compared to mammals, avian embryonic development is visible *in vitro* and relatively easy to manipulate. Thus, we explored the biological function of the PIWI protein and PIWI binding to small RNAs by using deep sequencing technology for the first time. Our findings will indicate the fundamental importance of recording the small RNA profile to understand gene regulation during spermatogenesis.

## Materials and Methods

### 1. Ethics statement

This study was carried out in strict accordance with the recommendations in the Guide for the Care and Use of Laboratory Animals of Jiangsu province. The protocol was approved by the government of Jiangsu Province, China (Permit Number: 45). All efforts were made to minimize suffering.

### 2. Sample collection and RNA isolation

Different tissues, including the heart, liver, kidney, brain, testis, ovary, pectoral muscle and lung, were obtained from adult Common quails (*Coturnix coturnix*) (45 d) from a quail breeding farm in Taizhou, Jiangsu Province, and stored at −70°C. Total RNA was extracted using the TRIzol reagent (Invitrogen), and the concentration was determined using a NanoDrop Spectrophotometer (Thermo Fisher Company).

### 3. Cloning of the quail *PIWI* gene

Using the mRNA sequence of the red jungle fowl (GeneID: 416804), the CDS of the *PIWIL1* gene was amplified from adult quail testis using the following primers: F-5′ATGACAGGAAGAGCTAGAGCC and R-5′TTAGAGATAGTAAAGTCTG. RACE was performed using the SMARTer™ RACE cDNA Amplification Kit (Clontech) with the GSP1 (5′-GGTAGCTCCCTTAATCCGTGCAAAGCC) and GSP2 (5′-CGAGCAATCACACACTGACTTGGAATGGGAC) primers.

### 4. Antibody generation and immunoprecipitation

A *PIWIL1* cDNA fragment corresponding to amino acids 697–867 was cloned into the pET-32a vector for bacterial overexpression of the peptide. The solubilized inclusion bodies were used to immunize two New Zealand rabbits. Antibodies were affinity purified by immunoadsorption (Shanghai Bio-ferry Biotechnology Co., Ltd.). Immunoprecipitation of the PIWIL1-RNA complex in adult quail testicular extract was performed using the PIWIL1 polyclonal antibody and the Magna RIP™ RNA-Binding Protein Immunoprecipitation Kit (Millipore). The binding RNA was prepared from the RNP complex.

### 5. Northern blotting

DNA probes complementary to the *PIWIL1* mRNA sequence were labeled with DIG-dUTP using the DIG DNA labeling kit (Shenzhen Labkit Bioscience Co., Ltd.). Total RNAs were fractionated on a 1.2% formaldehyde denaturing gel (25 µg/lane). After electrophoresis, the gel was transferred by capillary action with 20× SSC onto a nylon membrane (positively charged) following UV crosslinking (5,000 µJ/cm^2^). The probe/hybridization mixture (3 ng/ml) was added and incubated overnight with gentle agitation. After hybridization and stringency washes, the membrane was incubated for 30 min in blocking buffer according to the DIG detection Kit II (Shenzhen Labkit Bioscience Co., Ltd.). The membrane was incubated with anti-DIG-AP at a dilution of 1∶20,000 for 30 min at room temperature and washed with 0.1× SSC/0.1% SDS solution followed by incubation with CDP-STAR at a dilution of 1∶100 for 5 min at room temperature. The membrane was exposed to X-ray film for 1 min and fixed.

### 6. Western blotting

Tissues were homogenized in 3–5 volumes of lysis solution with PMSF (Beijing Dingguo Changsheng Biotechnology Co., Ltd.) and centrifuged at 13000× g for 5 min at 4°C (Eppendorf 5415R centrifuge). The concentration of the liquid supernatant was determined using the BCA method (Beijing Dingguo Changsheng Biotechnology Co., Ltd.). The total protein was separated by 10% SDS-PAGE (50 µg/lane) and electro-transferred to a PVDF membrane (300 mA, 1 h). The transferred membrane was rinsed and blocked with 5% BSA overnight at 4°C. The membrane was incubated with rabbit polyclonal anti-chicken PIWIL1 antibody at a dilution of 1∶8000 for 1.5 h at room temperature. After rinsing, the membrane was incubated with IgG anti-rabbit secondary antibody coupled to peroxidase (Wuhan Boster Bio-engineering Co., Ltd.) at a 1∶3,000 dilution for 1.5 h at room temperature. After a 10 min incubation with the chemiluminescent substrate, the membrane was exposed to film (Kodak).

### 7. Small RNA library construction and sequencing

The small RNA fraction between 18 and 30 nt was isolated by 15% denaturing PAGE and ligated with proprietary adaptors. The ligated RNA was then converted to cDNA by RT-PCR, and the cDNA was sequenced on Illumina Genome Analyzer II× following the protocol of the Illumina TruSeq Small RNA Preparation kit (Illumina, San Diego, USA). The initial sequencing results were converted into sequence data by base calling to generate raw data.

### 8. Bioinformatics analysis

The raw data underwent data cleaning, which included discarding the low quality reads and several kinds of contaminants. The identical reads in the raw data were clustered into unique families. By mapping clean reads to a reference genome (*Gallus gallus* v.2.1) and known databases, such as Rfam (10.1) and miRBase (18.0), small RNAs could be annotated into different categories. Those small RNAs that could not be annotated were used to predict novel miRNAs. The data analysis of small RNAs fraction of 23–32 nt was carried out according to the previous research method [13].

## Results

### Molecular cloning and localization of the quail *PIWIL1* gene

**Figure 1:**
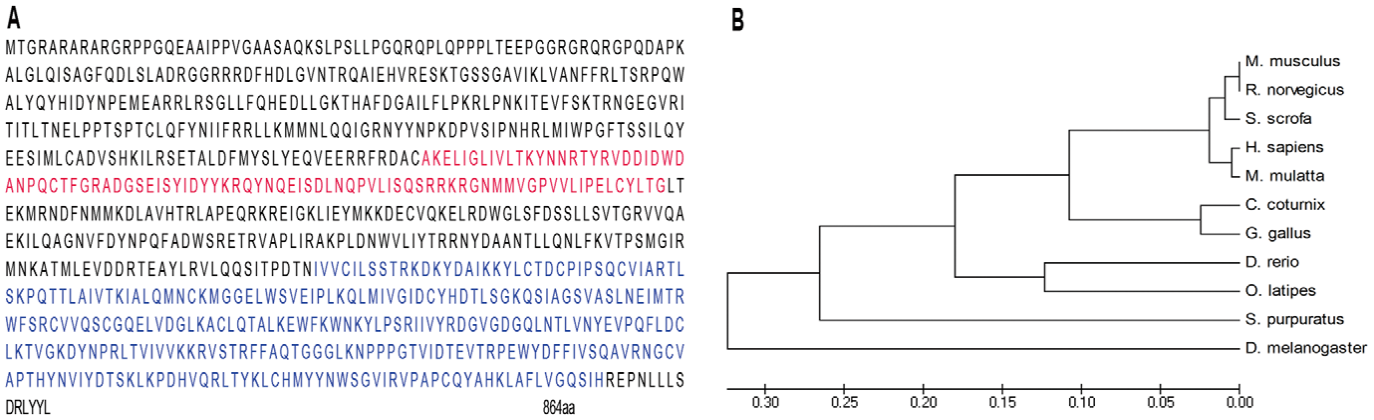
The PIWIL1 protein. A: Complete amino acid sequence of PIWIL1; PAZ domain, red; PIWI domain, blue. B: A phylogenetic tree of the PIWIL1 protein generated using UPGMA.

To investigate the function of the quail PIWI protein, we isolated a 3.4 kb *PIWI* cDNA from adult testis containing a 142 bp 5′ untranslated region (UTR), a 2.59 kb open reading frame (ORF) and a 667 bp 3′ UTR followed by a poly(A) tail and named it *PIWI*-like 1, or *PIWIL1*. The ORF predicted that the encoded PIWIL1 protein contained 864 amino acid residues (Figure 1A) with a relative molecular mass of 98,000 and an isoelectric point of 9.33. The C-terminal PIWI domain (558–850 aa) and the 110-amino acid PAZ domain (305–394 aa) were also detected, suggesting that it is a potential member of the PIWI protein family. Additionally, the conserved homology is particularly high between different species (Figure 1B).

**Figure 2:**
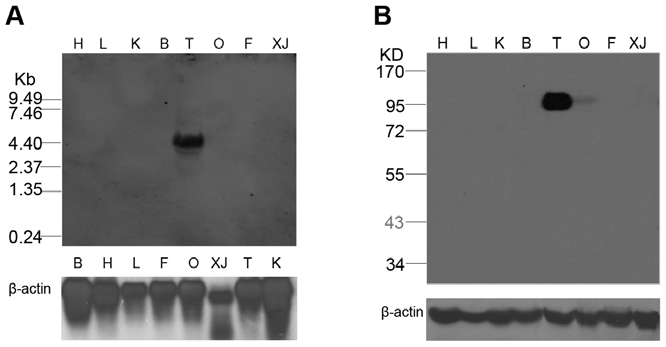
*PIWIL1* is specifically expressed in the testis. A: Northern blot analysis of *PIWIL1* expression in adult tissues with *β-actin* as the loading control. B: Western blot analysis of adult tissues; H, heart; L, live; K, kidney; B, brain; T, testis; O, ovary; F, lung; XJ, pectoral muscle.

To explore the role of *PIWIL1* in development, we examined its expression in major adult organs by Northern blotting (Figure 2A). The *PIWIL1* gene encoded a 4.4 kb transcript that was specifically expressed in the adult testis. The adult- and testis-specific expression of *PIWIL1* was further confirmed by Western blotting (Figures 2B and S1). As expected, a 98 kDa protein fragment was detected in the adult testis. And it was indicated that our anti-PIWIL1 antibody could recognize the PIWIL1 protein from the testicular extract.

### An overview of Solexa sequencing

**Figure 3:**
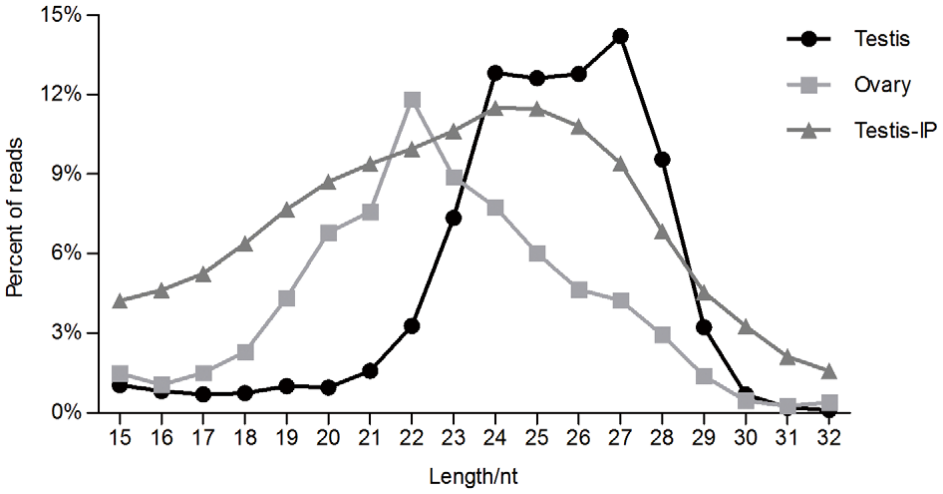
Length distribution of small RNAs. The ratio of small RNAs was magnified 1.7 times from the IP product.

In order to identify the small RNAs that are relevant in quail spermatogenesis, the PIWIL1-RNA complex in adult testicular extract was obtained by immunoprecipitation (IP), and the small RNA library was constructed. Simultaneously, the small RNA fraction (18–30 nt) was isolated from total RNA in adult quail testis and ovary. We obtained 21,131,183 reads from the IP product, 11,350,879 reads from the testis and 10,495,986 reads from the ovary. The ovarian RNA library showed a peak at 22 nt corresponding to miRNAs (Figure 3). The testicular RNA library demonstrated a bimodal distribution with two strong peaks at 24 and 27 nt, and PIWIL1 binding to small RNAs showed a weak peak at 24–25 nt.

### Mapping and cataloging miRNAs expressed in the adult quail testis and ovary

We obtained 15,917,971 (75.33%), 11,121,041 (97.98%) and 8,190,484 (78.03%) clean reads from the IP product, testis and ovary, respectively. Using BLAST to search against the *Gallus gallus* genome and miRBase (18.0), miRNAs were identified from all three small RNAs libraries and divided into seven groups in order of high-to-mid confidence (Table 1). In the deep sequencing data, we frequently observed sequence heterogeneity at the 5′ and 3′ ends, which demonstrated that multiple mature variants called isomiRs were produced. Generally, the most abundant isomiR was chosen as a reference sequence to provide the most robust approach to evaluate the differential expression of miRNAs. In the ovarian RNA library, we detected 311 unique miRNAs corresponding to 212 known *Gallus gallus* pre-miRNA, which represents less than half of the 499 pre-miRNAs in miRBase (18.0) depository. However, the ratio of all known miRNA copies in the ovarian RNA library ranged up to 17.7% (1.9% for the testicular RNA library and 0.1% for the IP product), suggesting that miRNAs were the dominant species of small RNA (Table S1).

**Table 1:** Pre-miRNAs and mature miRNAs identified in three libraries.

Group	Testis		Ovary		Testis-IP	
	miRs#	mirs#	miRs#	mirs#	miRs#	mirs#
gp1a^a^	238	169	341	212	195	157
gp1b^b^	18	12	32	22	20	13
gp2a^c^	11	11	28	26	4	4
gp2b^d^	69	53	122	111	66	64
gp3a^e^	220	192	490	397	282	251
gp3b^f^	375	340	261	218	93	84
gp4a^g^	951	844	1868	1735	2525	2415

To investigate the differential expression of the miRNAs, we normalized and compared all unique miRNAs using IDEG6. The miRNA counts showed a significant difference between the testicular and ovarian RNA libraries (Table S3). For example, the gga-miR-148a count was 146 in the testicular RNA library, and it was up to 19,754 in the ovarian RNA library. The miRNA transcriptome of the quail gonads consisted of unevenly distributed counts of sequences. We found that most of the top ten miRNAs in the testis were from the let-7-family of miRNAs. Compared to the testis, gga-let-7a, gga-let-7f and gga-let-7c were down-regulated in the ovary, while gga-miR-26a, gga-miR-148a and gga-miR-451 were up-regulated in the ovary. However, the top ten miRNAs in the testis and ovary were down-regulated in the RNA library from the immunoprecipitated product.

### Identification of rasiRNAs in the quail

In the testicular RNA library, the length distribution of small RNAs revealed a strong peak at 24–27 nt, similar to rasiRNAs and piRNAs. To identify the genetic characteristics of these small RNAs in quail, the sequences in the 23–32 nt range that mapped to the *Gallus gallus* genome were analyzed and found to contain 566,212 (4.99%), 928,311 (8.84%) and 690,891 (3.27%) clean reads from the testis, ovary and IP product, respectively. As shown in Table S2, 8.68% (testis), 22.74% (ovary) and 5.41% (IP product) of unique reads were annotated as previously known non-coding RNAs (Table S2). It had been suggested that miRNAs were not a major component of the testicular RNA library. A less extreme 5′ nucleotide bias for uracil was observed in the remainder of the unique reads (Figure S2). In the testicular RNA library, approximately 36% of unique reads mapped to repeat sequences including LINE, LTR, satellites and other repeat sequences (Table S2). In the IP product library, up to 81.44% of unique reads mapped to the *Gallus gallus* genome for one hit, 12.25% of unique reads were located in repeat sequences and 14.86% of unique reads corresponded to exons.

**Figure 4:**
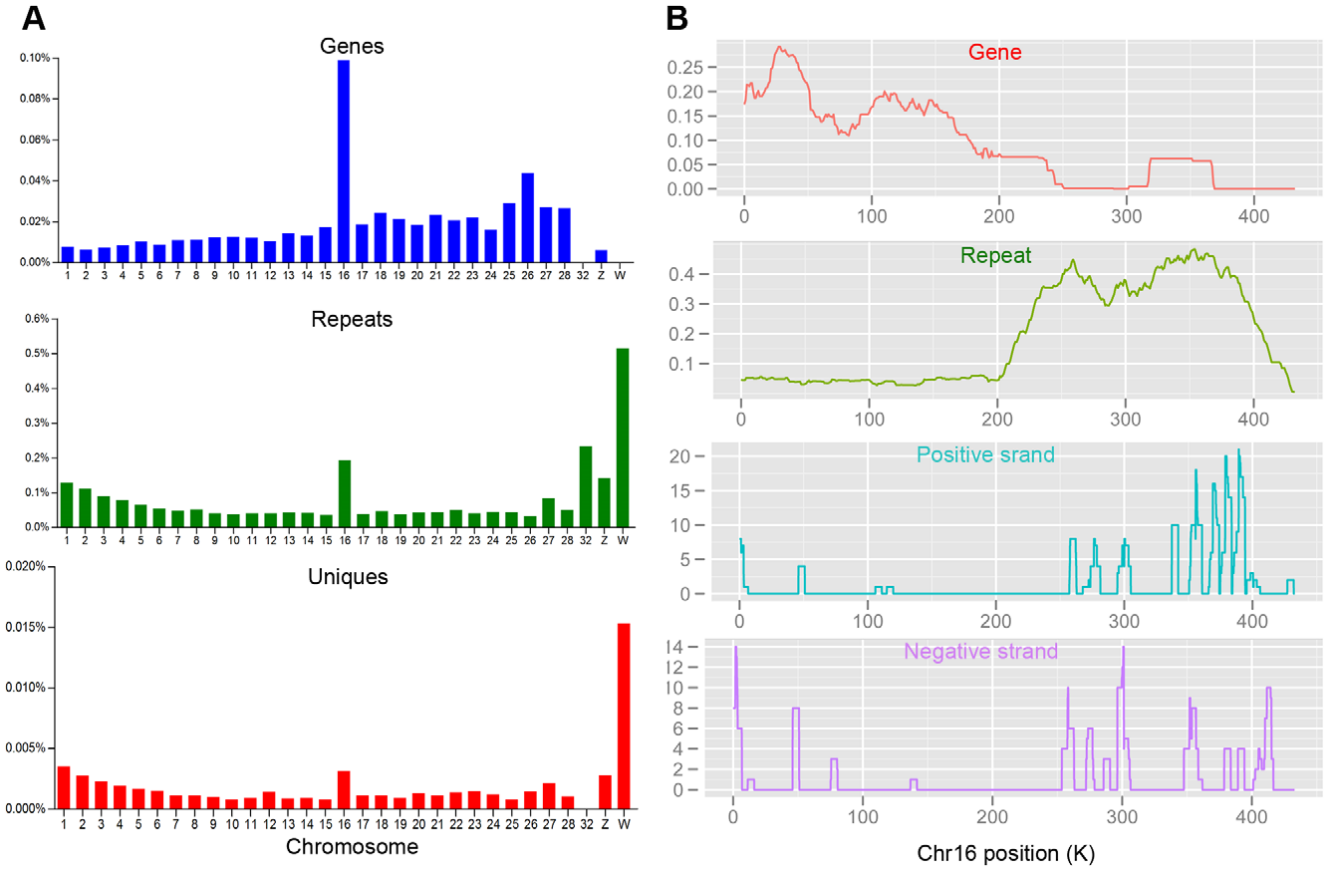
Properties of unannotated unique reads in the testis. A: Chromosomal distribution of exons (top), repeat sequences (middle) and unannotated unique reads (bottom). The number of bases in exons of Refseq genes and repeats are plotted as a percentage of overall chromosome length. The numbers of piRNAs are normalized to chromosome length and plotted. B: Density analysis of exons (red), repeat sequences (green) and unannotated unique reads (with the positive strand in blue and the negative strand in purple) along chromosome 16. The densities of exons and repeats were using a 50 kB window, scanning the genome in increments of 1000 bases. Unannotaed uniques densities were determined by calculating a moving average of reads in a 5 kB sliding window (100 base increments) along each chromosome. Only reads that map 1 to 5 times to the genome were used in density analysis.

An examination of the unannotated unique reads that mapped to 1–5 discrete loci revealed a non-uniform distribution in the genome (Figures 4A, S3, and S4). Unannotated unique reads were primarily found in chromosome W, followed by chromosomes Z and 16, which is consistent with the distribution of the repeat sequences. The majority of the coding sequences are concentrated in chromosome 16, and only a small number are found in chromosome Z. There are no coding sequences in chromosome W. We propose that the unannotated unique reads came primarily from repeat sequences followed by genic sequences. Correspondingly, the most unannotated unique reads were located in the repeat sequences on chromosome 16, and few unannotated unique reads were derived from chromosome 16 genes (Figure 4B). A detailed view revealed the distribution of unannotated unique reads along both strands of the genome, which differed from the single stranded-bias of piRNAs in mammals.

## Discussion

### *PIWIL1* is specifically expressed in the quail testis

In Drosophila, *PIWI* encodes a nucleoplasmic protein whose activity modulates the number and division rate of germline stem cells [2]. In mice, the PIWI protein is expressed only in male germ cells. MIWI and MILI are cytoplasmic proteins [9]–[10], and MIWI2 is expressed in the nucleus for short-term [11]. In this study, we cloned a 3.4 kb cDNA from adult quail testis that encodes an 864 amino acid protein with PAZ and PIWI domains. Additionally, the CDS sequence shares significant homology with other PIWI proteins. The PIWIL1 protein is specifically expressed in the adult testis, which is similar to the MIWI protein in mice [9] and suggests that PIWIL1 might be involved in spermatogenesis. Spermatogonial development in quail differs from other mammals, as there are fewer mitotic divisions and they are synchronized with the cell cycle of the seminiferous epithelium [26]. If the PIWIL1 protein is involved in spermatogenesis, then the molecular mechanisms that are responsible for the differences between quail and other mammals require further study.

### PIWIL1 binds to quail rasiRNA

In mammals, the PIWI protein and piRNAs are RISC components that regulate spermatogenesis. To determine the mechanism of PIWI-mediated piRNA pathway in quail, we obtained the PIWIL1-RNA complex from testicular extract of adult quail and purified the associated RNAs. The length distribution of PIWIL1 binding to small RNAs showed a predominant peak at 24–25 nt, which was similar to the length distribution of rasiRNAs in Drosophila [6] and shorter than that of piRNAs in mice [13]. To identify the genetic characteristics of the small RNAs bound by PIWIL1, we mapped our sequences (23–32 nt) to known non-coding RNAs databases and the *Gallus gallus* genome. Given the unique peak at 24–27 nt in the testicular RNA library, very few unique reads were annotated, suggesting that the remaining reads likely belonged to a novel class of small RNAs. However, a less extreme 5′ uracil bias had been noted in unknown unique reads, which differed from the extreme 5′ uracil bias for piRNAs [13]. In mammals, piRNAs were mainly distributed in the intergenic sequences and had only one hit in the genome, while our unknown unique reads mapped to the *Gallus gallus* genome more than once. The unique reads were mainly concentrated in chromosome W followed by chromosome Z from the testis and ovary, which was consistent with the distribution of repeat sequences. It appeared that the unknown unique reads may be involved in the transcription or translation of repeat sequences in the germline. Given the nucleotide length, testicular specificity, PIWI protein interaction and genomic origin, we hypothesized that the unknown unique sequences were quail rasiRNAs. However, many of the unknown unique sequences from the testis mapped to chromosome W in our study. At present, we are unable to explain this phenomenon. Whether there is a relationship between PIWIL1 binding to small RNAs and the sex determination mechanism requires more experimental evidence.

### PIWIL1 is associated with quail miRNAs

As we all known, the PIWI subfamily protein plays an important role in the germline but its molecular mechanism remains unclear. It was reported that the functional role of PIWI protein in Drosophila germline determination is due to their capacity to interact with miRNAs [27]. The PIWI-mediated miRNA pathway positively regulates the expression of Drosophila germline genes such as *OSK* and *VASA*, which differs from the known translational repression role of the miRNA pathway. In support of this point, MIWI also appears to positively regulate gene expression in mice [9]. In our study, some of known miRNAs were detected from the IP product library, suggesting that miRNAs interact with the PIWIL1 protein and are likely to play a role in the quail testis. And the expression of these miRNA-targeted genes will be our next study work.

### miRNAs are differentially expressed between adult testis and ovary

Reproduction-related miRNAs have been the recent subject of intense interest. Sexually dimorphic miRNA expression has been reported during the chicken embryonic gonadal development, suggesting that miR-202* may function in regulating testicular development [28]. In our study, we analyzed the differential expression of miRNAs in the adult quail gonad and found that the expression of miR-202* had no difference in both sexes. But the expression miR-202 in adult testis was significantly higher than that in adult ovary, hinting that miR-202 maybe play a role in adult testis. Let-7 family miRNAs are ubiquitously expressed in various cell and tissues types at high levels and are involved in the cell cycle as master regulators of cell proliferation pathways [29]–[31]. The relative abundance of these miRNAs in the testis and ovary suggests that they may play a role in testicular and ovarian development. Other recent studies also support our results. For example, let-7a, let-7b and let-7i were shown to be up-regulated, while let-7f and miR-21 were shown to be down-regulated in human epithelial ovarian cancer [32]. As determined by in situ hybridizations, miR-21 and let-7a were found at high concentrations in the perinuclear granules in round spermatids [33]. Let-7f and let-7i were down-regulated in human non-obstructive azoospermia [34]. These findings support the relevance of miRNAs in ovarian and testicular physiology and indicate that they may be the signature of disease in gonads. Consequently, we intend to further explore the function of differential miRNAs.

## Supporting Information

Figure S1 **Immunoprecipitation.** The native PIWIL1 protein was captured by the anti-PIWIL1 antibody from the adult testicular lysate and then detected by Western blot (Lane 1). Brain served as the PIWIL1-negative control (Lane 2). Pre-immune serum from rabbits (purified) served as the antibody-negative control (Lane 3). The adult testicular lysate served as the PIWIL1-positive control (Lane 4).
(TIF)

Figure S2 **Nucleotide bias of the small RNAs.** For each position, the proportion of A, U, G and C found in the unannotaed unique reads is represented.
(TIF)

Figure S3 **Chromosome distribution of unannotated unique reads in the ovary.** The number of bases in exons of Refseq genes and repeats are plotted as a percentage of overall chromosome length. The numbers of piRNAs are normalized to chromosome length and plotted.
(TIF)

Figure S4 **Chromosome distribution of unannotated unique reads in the IP product.** The number of bases in exons of Refseq genes and repeats are plotted as a percentage of overall chromosome length. The numbers of piRNAs are normalized to chromosome length and plotted.
(TIF)

Table S1 **Distribution of small RNA among different categories in three libraries (miRNAs analysis).**
(DOC)

Table S2 **Distribution of small RNA among different categories in three libraries (rasiRNAs analysis).**
(DOC)

Table S3 **Differential expression of miRNAs in testis and ovary.** All unique miRNAs from testis and ovary libraries were normalized and used to detect their differential expression using IDEG6.
(XLS)
