# Supplementary figures and images for: Cloning of the Quail PIWI Gene and Characterization of PIWI Binding to Small RNAs
Source: PLoS One. 2012 Dec 19;7(12):e51724. doi: 10.1371/journal.pone.0051724 (PMC3526641; doi:10.1371/journal.pone.0051724)

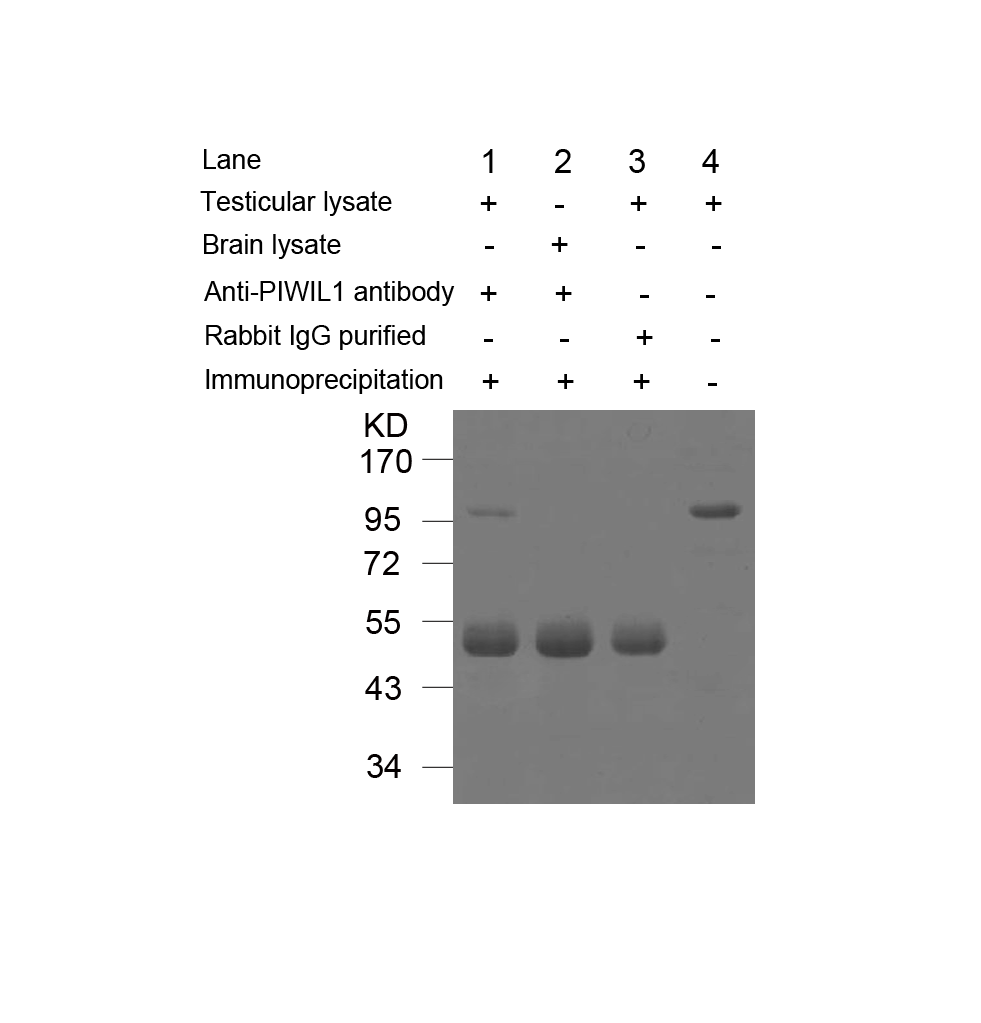

Supplement: Figure S1 — Immunoprecipitation. The native PIWIL1 protein was captured by the anti-PIWIL1 antibody from the adult testicular lysate and then detected by Western blot (Lane 1). Brain served as the PIWIL1-negative control (Lane 2). Pre-immune serum from rabbits (purified) served as the antibody-negative control (Lane 3). The adult testicular lysate served as the PIWIL1-positive control (Lane 4). (TIF) [file pone.0051724.s001.tif]

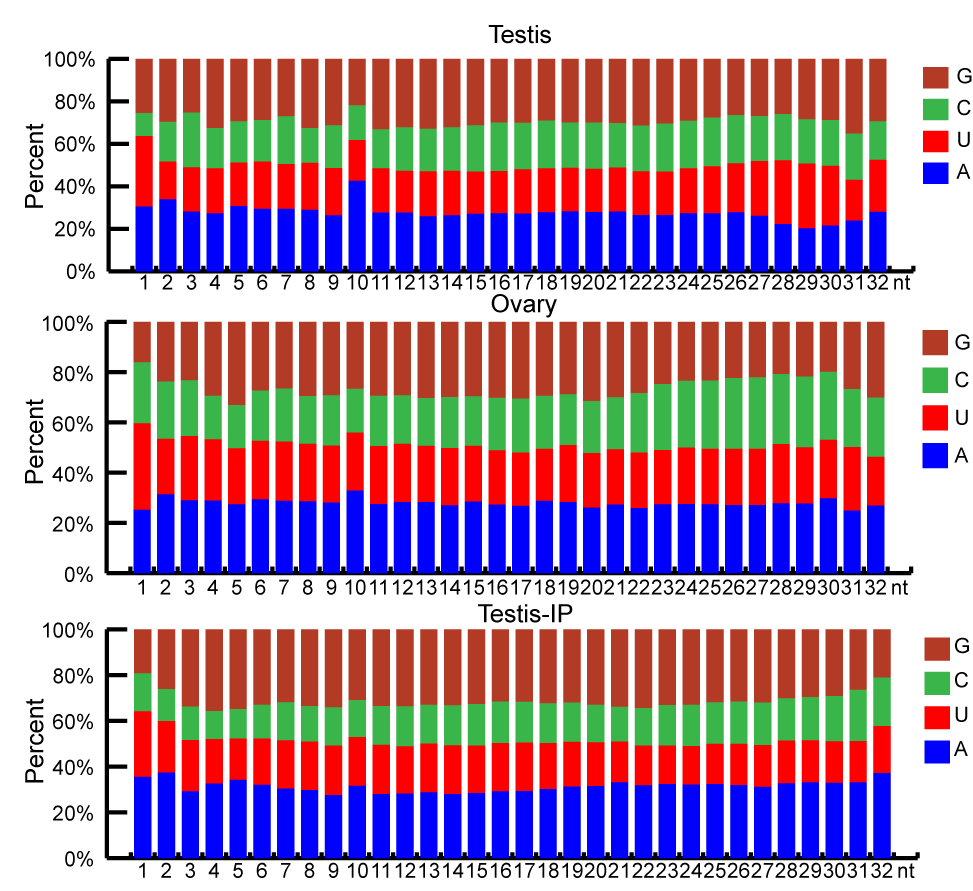

Supplement: Figure S2 — Nucleotide bias of the small RNAs. For each position, the proportion of A, U, G and C found in the unannotaed unique reads is represented. (TIF) [file pone.0051724.s002.tif]

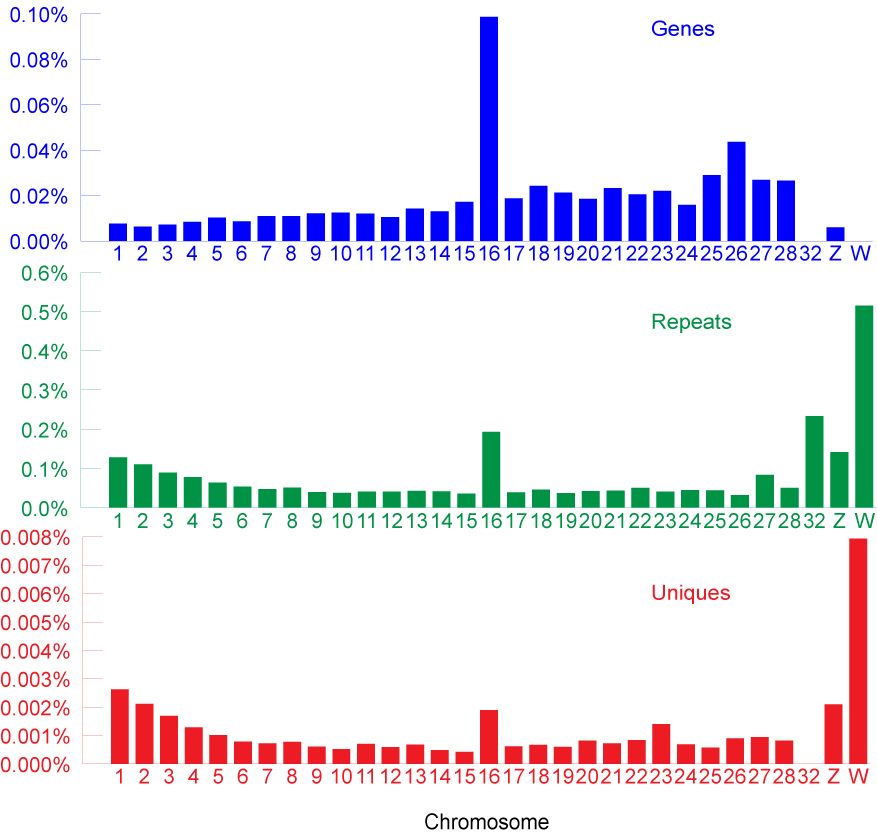

Supplement: Figure S3 — Chromosome distribution of unannotated unique reads in the ovary. The number of bases in exons of Refseq genes and repeats are plotted as a percentage of overall chromosome length. The numbers of piRNAs are normalized to chromosome length and plotted. (TIF) [file pone.0051724.s003.tif]

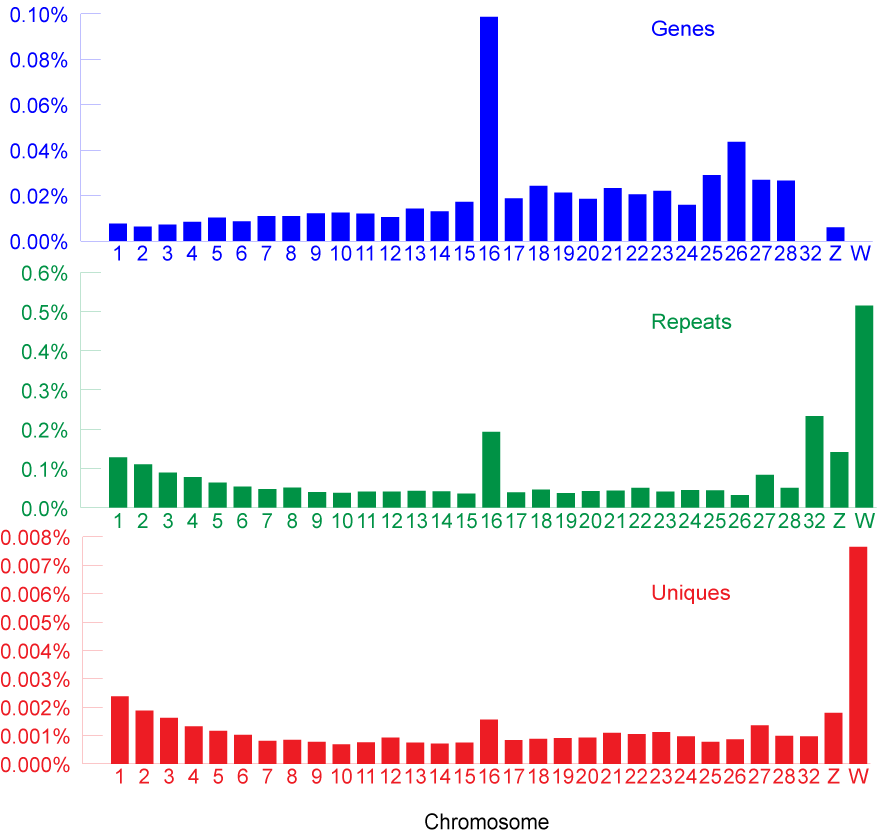

Supplement: Figure S4 — Chromosome distribution of unannotated unique reads in the IP product. The number of bases in exons of Refseq genes and repeats are plotted as a percentage of overall chromosome length. The numbers of piRNAs are normalized to chromosome length and plotted. (TIF) [file pone.0051724.s004.tif]
