# Supplementary material for: Cloning of the Quail PIWI Gene and Characterization of PIWI Binding to Small RNAs
Source: PLoS One. 2012 Dec 19;7(12):e51724. doi: 10.1371/journal.pone.0051724 (PMC3526641; doi:10.1371/journal.pone.0051724)
Supplement: Table S1 — Distribution of small RNA among different categories in three libraries (miRNAs analysis). (DOC) [file pone.0051724.s005.doc]

Table S1 Distribution of small RNA among different categories in three libraries

(miRNAs analysis)

| Types | Testis | | Ovary | | Testis-IP | |
| --- | --- | --- | --- | --- | --- | --- |
|  | Reads# | % | Reads# | % | Reads# | % |
| Total | 11,121,041 | 100.0 | 8,190,484 | 100.0 | 15,917,971 | 100.0 |
| gp1a[[1]](#footnote-2) | 318,729 | 2.9 | 1,452,097 | 17.7 | 16,900 | 0.1 |
| gp1b[[2]](#footnote-3) | 2,208 | 0.0 | 13,635 | 0.2 | 4,011 | 0.0 |
| gp2a[[3]](#footnote-4) | 107 | 0.0 | 785 | 0.0 | 128 | 0.0 |
| gp2b[[4]](#footnote-5) | 323 | 0.0 | 1,424 | 0.0 | 525 | 0.0 |
| gp3a[[5]](#footnote-6) | 6,586 | 0.1 | 110,978 | 1.4 | 8,233 | 0.1 |
| gp3b[[6]](#footnote-7) | 1,538 | 0.0 | 10,811 | 0.1 | 837 | 0.0 |
| gp4a[[7]](#footnote-8) | 5,920 | 0.1 | 5,187 | 0.1 | 10,861 | 0.1 |
| gp4b[[8]](#footnote-9) | 398,454 | 3.6 | 205,457 | 2.5 | 443,367 | 2.8 |
| mRNA | 461,905 | 4.2 | 1,142,056 | 13.9 | 1,340,058 | 8.4 |
| Rfam | 84,653 | 0.8 | 427,227 | 5.2 | 1,465,384 | 9.2 |
| Repbase | 36,832 | 0.3 | 57,041 | 0.7 | 27,565 | 0.2 |
| Nohit | 9,883,373 | 88.9 | 5,001,948 | 61.1 | 12,792,188 | 80.4 |

1. known miRs of Gallus gallus [↑](#footnote-ref-2)
2. known miRs of other vertebrate [↑](#footnote-ref-3)
3. mapped to known mirs of other vertebrate and genome with hairpins [↑](#footnote-ref-4)
4. mapped to known mirs of other vertebrate and genome without hairpins [↑](#footnote-ref-5)
5. mapped to known mirs and miRs of other vertebrate but unmapped to genome [↑](#footnote-ref-6)
6. mapped to known mirs of other vertebrate but unmapped to genome [↑](#footnote-ref-7)
7. unmapped to known mirs but mapped to genome with hairpins [↑](#footnote-ref-8)
8. unmapped to known mirs but mapped to genome without hairpins [↑](#footnote-ref-9)
