# Supplementary material for: Cloning of the Quail PIWI Gene and Characterization of PIWI Binding to Small RNAs
Source: PLoS One. 2012 Dec 19;7(12):e51724. doi: 10.1371/journal.pone.0051724 (PMC3526641; doi:10.1371/journal.pone.0051724)
Supplement: Table S2 — Distribution of small RNA among different categories in three libraries (rasiRNAs analysis). (DOC) [file pone.0051724.s006.doc]

Table S2 Distribution of small RNA among different categories in three libraries

(rasiRNAs analysis)

| type | Testis | | Ovary | | Testis-IP | |
| --- | --- | --- | --- | --- | --- | --- |
|  | Uniques | Reads | Uniques | Reads | Uniques | Reads |
| total | 130111 | 566212 | 87990 | 928311 | 183686 | 690891 |
| uniq | 72665 | 231590 | 61793 | 431910 | 149588 | 506762 |
| no_uniq | 57446 | 334622 | 26197 | 496401 | 34098 | 184129 |
| genic | 20601 | 184287 | 32120 | 716846 | 46629 | 333765 |
| intron | 5030 | 19217 | 3417 | 5196 | 9396 | 23633 |
| exon | 4276 | 9545 | 8694 | 10850 | 27291 | 59113 |
| miRNA | 380 | 25460 | 617 | 243404 | 266 | 4598 |
| rRNA | 9207 | 116754 | 15145 | 343115 | 7728 | 229237 |
| tRNA | 867 | 11350 | 1682 | 102116 | 418 | 4168 |
| scRNA | 178 | 483 | 387 | 2347 | 80 | 369 |
| snRNA | 545 | 1157 | 1119 | 3157 | 719 | 8057 |
| snoRNA | 118 | 321 | 1059 | 6661 | 731 | 4590 |
| repeat | 46968 | 105643 | 20462 | 98787 | 22493 | 59473 |
| LTR | 6580 | 16533 | 3568 | 14229 | 2463 | 6557 |
| LINE | 36780 | 78748 | 16104 | 82935 | 18471 | 48906 |
| Satellite | 827 | 5145 | 83 | 111 | 485 | 1425 |
| other | 2781 | 5217 | 707 | 1512 | 1074 | 2585 |
